# Antimicrobial, Cytotoxic and Oxidative Stress Inhibitory Activities of Terpenoids and Flavonols from *Senegalia nigrescens* (Oliv.) P.J.H. Hurter

**DOI:** 10.22037/ijpr.2021.115653.15463

**Published:** 2021

**Authors:** Olusola Bodede, Garland K. More, Gerhard Prinsloo

**Affiliations:** *Department of Agriculture and Animal Health, University of South Africa, Florida Campus, Florida, 1710, South Africa.*

**Keywords:** Antimicrobial activity, Anticancer activity, Oxidative stress, ent-kaurene, Quercetin

## Abstract

*Senegalia nigrescens* (knob thorn) is a deciduous tree distributed in savannah regions from Tanzania to South Africa used for timber but also medicinally for the treatment of convulsions, wounds, and skin problems. In this study, the biological activities of six phytocompounds, namely: 3***β***-hydroxy-20(29)-en-lupan-30-al (**1**), 30-hydroxylup-20(29)-en-3***β***-ol (**2**), *ent*-kaur-15-en-18,20-diol (**3**), melanoxetin (**4**), quercetin (**5**) and quercetin-3-*O*-methyl ether (**6**), isolated from *S. nigrescens* were investigated. The compounds were screened against two bacterial (*Escherichia coli* and *Staphylococcus aureus*) and one fungal (*Candida albicans*) strain and were also tested for their cytotoxicity on breast cancer (MDA-MB-231) and normal murine macrophage (RAW 264.7) cell line. Effects of the compounds on attenuating the lipopolysaccharide (LPS)-induced intracellular reactive oxygen species (ROS) production in RAW 264.7 cells were quantified with the H_2_DCF-DA assay. This study revealed that flavonols (**5** and **6**) had the strongest antibacterial and antifungal effects, both having MIC values of 62.5, 31.25 and 31.25 µg/mL on *E. coli*, *S. aureus* and *C. albicans*, respectively. Compounds **2**, **3** and **6** were the most cytotoxic against the breast cancer cells with IC_50_ values of 11.86, 12.62 and 14.03 µg/mL, respectively, while the least toxicity towards normal cells were observed in compounds **2**, **5** and **6**. All compounds (**1**-**6**) significantly lowered ROS production in RAW264.7 cells. In conclusion, tested compounds represent potential promising candidates as antimicrobial, anticancer and antidotes for LPS-induced oxidative stress. This is the first report on the antifungal, cytotoxicity and antioxidative activities of the *ent*-kaurene diterpenoid, *ent*-kaur-15-en-18,20-diol (**3**).

## Introduction

Globally, microorganisms such as viruses, bacteria, and fungi pose challenges to public health especially in the advent of drug-resistant strains. In recent years, microbial resistance to drugs has attracted considerable attention from researchers in different fields of research including pharmaceutical, food safety and climate change industries, to mention a few. In food industries, *E. coli, Listeria, Campylobacter*,* Staphylococcus *and* Salmonella *species, and yeast have been reported as the predominant causative agents of foodborne diseases (1). *Staphylococcus, E. coli, Klebsiella pneumoniae, Acinetobacter *species, and* Candida* species have been implicated to contribute to most common healthcare-associated infections (2, 3). However, because of the increasing pervasiveness of drug-resistant bacterial strains, there is a demand to develop alternative antimicrobial agents to control infections and the spread of diseases. Other degenerative diseases have also increased in recent years and these include cancer, diabetes, atherosclerosis, rheumatoid arthritis, Parkinson’s disease (PD), and Alzheimer’s disease to mention a few. 

Cancer is one of the most deadly diseases characterized by cells multiplying continuously spreading malignant cells forming tumors with a possibility of becoming metastatic. During infection, phagocytic cells play an important role in defending the immune system from inflammation and the spread of infections. Through their pattern recognition receptors, phagocytic cells respond to infection, injury or inflammation by secreting various proinflammatory mediators, including tumor necrosis factor-α (TNF-α) and interleukin-1β (IL-1β), prostaglandin (PG) E_2_ as well as the free radicals, nitric oxide (NO) and reactive oxygen/nitrogen species (ROS/RNS) (4). The presence of ROS has been linked to many biological processes in normal and cancer cells and these processes include cell signaling (5). They have been said to be two-faced in their cellular functions with many studies highlighting the functions of ROS as tumor-promoting or tumor-suppressing mediators (6, 7) with high amounts of ROS contributing to malignant diseases (8). Maintenance of intracellular balance is necessary to prevent the accumulation of ROS which leads to disease progression. This intracellular defense involves both enzymatic and non-enzymatic antioxidants. Many studies have shown that antioxidants are beneficial for both the prevention and treatment of cancer because they can quench ROS (8). However, these compounds (at certain concentrations or under certain circumstances) may act as prooxidants (9, 10). Despite the d evelopment of treatments (chemotherapy, radiation hormonal therapy) for cancer, it has been reported that patients on chemotherapy experience some adverse effects which may further mutilate their health. In continuation of our studies on *S. nigrescens* (formerly *Acacia nigrescens*) (11-13), the biological activities of six *S. nigrescens *phytocompounds for their antimicrobial, anticancer activity on human breast cancer cell line (MDA-MB-231) and normal murine macrophage cell line (RAW264.7) are reported, and further assessed their suppression of LPS induced oxidative stress. 

## Experimental

*Chemicals, reagents and cells*

Solvents (analytical grade) and other chemicals used for the chemistry work were supplied by either Merck (Darmstadt, Germany) or Sigma (St. Louis, MO, USA) chemical companies. Murine macrophage cells (RAW264.7) and breast adenocarcinoma‎ cells (MDA-MB-231) were purchased from Cellonex Separation Scientific SA (Pty) Ltd. (Roodepoort, Johannesburg, South Africa). The Dulbecco’s modified eagle’s medium, fetal bovine serum, and penicillin-streptomycin were bought from Celtic Molecular Diagnostics SA (Pty) Ltd. (Cape Town, South Africa). Dimethyl sulfoxide (DMSO), 3-(4,5-dimethylthiazol-2-yl)-2,5-diphenyltetrazolium bromide (MTT), Nutrient agar, Nutrient broth, Sabouraud Dextrose Agar (SDA), Sabouraud dextrose broth (SDB), Ciprofloxacin, Amphotericin-B, Resazurin sodium salt, Doxorubicin, Lipopolysaccharide (LPS) and 2',7'-dichlorodihydrofluorescein diacetate, were purchased from Sigma-Aldrich®, (St. Louis, MO, USA) and Merck (Darmstadt, Germany).

*Microbial strains and Microorganisms maintenance*

Three microorganisms used in this study, two bacterial strains (*Escherichia coli* ATCC 25922 - Gram-negative and *Staphylococcus aureus* ATCC 25923 - Gram-positive) and a fungus (*Candida albicans* ATCC 10231) were procured from Anatech Instruments (Pty) Ltd, Randburg, Gauteng, South Africa (Anatech, Kwik-stick®). *Escherichia coli* and *S. aureus* were cultured in nutrient agar while the fungus (*C. albicans* ATCC 10231) was cultured on sabouraud dextrose agar (SDA) and incubated for 24 h under aerobic conditions at 37 °C. Pure colonies of microorganisms from the agar were transferred onto nutrient broth and sabouraud dextrose broth (SDB) to obtain an overnight culture. 

*Plant material*

As earlier reported (11), leaves, stem bark, and root of *S. nigrescens* were collected from the University of KwaZulu-Natal (Westville Campus) in January 2015. Authentication of the plant’s identity was done by Dr. Syd Ramdhani at the School of Life Sciences, University of KwaZulu-Natal. The plant’s voucher specimen with the number “Bodede 02” was deposited at the University’s Ward Herbarium.

*Compounds isolation*

The procedure for the isolation of compounds **1** – **6** was previously described (11). Briefly, non-polar (combined n-hexane and dichloromethane (DCM)) extract of *S. nigrescens* stem bark was subjected to column chromatography (CC) using gradient elution (n-hexane:ethyl acetate (EtOAc)) which ran from 10:0 to 0:10. One hundred aliquots of 100 mL each were obtained and combined into 6 fractions based on thin layer chromatography (TLC) analysis. The third and fourth fractions yielded **1** and **2**, respectively. Compound **3** was obtained from DCM extract of the root, following a similar chromatographic procedure described for **1** and **2**, above. A similar procedure was carried out on the ethyl acetate extract of the stem bark, which gave rise to 55 aliquots from which aliquots 35-43 were further purified on preparative TLC to give compound **4**. Compound **5** was obtained by slight modification of the chromatographic procedure described above. The methanol (MeOH) extract of the leaves was partitioned between DCM and EtOAc. The resulting EtOAc fraction was subjected to CC using n-hexane:EtOAc and EtOAc:MeOH solvents systems, starting from 100% n-hexane and finishing with 9:1 of EtOAc:MeOH. The fifteenth aliquot amongst the thirty aliquots collected yielded **5**. The EtOAc extract of the root was chromatographed using a similar approach as described above to obtain compound **5**. Thirty aliquots were collected and aliquot 20 yielded compound **6**. 

*Sample preparation*

Compounds **1** – **6** were weighed and dissolved in 5% Dimethyl sulfoxide (DMSO) to a final working stock solution of 4 mg/mL. Standard antibiotics Ciprofloxacin and Amphotericin-B were also dissolved in 5% DMSO. The samples were kept at 20 °C in the dark until used.

*Antimicrobial susceptibility test*

*Antimicrobial Assay*

Antimicrobial activity was done using the microplate serial dilution method by Eloff, (14). The minimum inhibitory concentration (MIC) was defined as the lowest concentration of the compound capable of inhibiting microbial growth. The microorganisms were adjusted according to 0.5 McFarland standard solution to a final concentration of 1.5 × 10^4^ CFU/mL in sterile 0.85% sodium chloride (NaCl) solution. Compounds were tested at the concentration range of 7.8-1000 µg/mL in 96 well plates and incubated with microorganisms for 24 h at 37 °C. Ciprofloxacin and Amphotericin-B were used as positive controls for bacterial and fungal assays, respectively. In addition, sterile distilled 5% DMSO was used as a negative control. A staining reagent, 50 µL Resazurin sodium salt (0.2 mg/mL) was used as an indicator of microbial growth and the MIC was visually determined (15).

*Cytotoxicity assay*

Cytotoxicity and antiproliferative effects of compounds were evaluated against Murine macrophage cells (RAW264.7) and breast adenocarcinoma‎ cancer cells (MDA-MB-231) using the spectrophotometric, 3-(4,5-dimethylthiazol-2-yl)-2,5-diphenyltetrazolium bromide (MTT) assay (16). Cells (5 × 10^4^ cell/well) were seeded in Dulbecco’s modified eagle’s medium (DMEM; Gibco) in a 96 well plate for 24 h incubation at 37 °C in 5% CO_2_ and treated with compounds (2.5 - 200 µg/mL) and positive control, Doxorubicin (2.5 - 200 µM). Untreated cells and 5% DMSO treated cells were assayed as negative and solvent control, respectively. After 24 h incubation, 20 µL MTT dye (5 mg/mL) was pipetted in all wells including wells with untreated cells, and incubated for 4 h. The medium was aspirated and 100 µL of DMSO was added to all the wells to dissolve the formazan crystals and the optical density (OD) was measured after an hour at 570 nm using a microplate reader. 

*Effects of compounds on intracellular ROS production in RAW264.7*

Lipopolysaccharide (LPS)-induced reactive oxygen species (ROS) production was probed using an oxidative cell-permeant fluorescent dye 2',7'-dichlorodihydrofluorescein diacetate (H_2_DCF-DA) (17). RAW264.7 cells (5 × 10^4 ^cells/well) were seeded overnight into a 96 well plate and treated with compounds (10 µg/mL) at concentrations ≤ IC_50_ values and LPS (10 µg/mL) for 24 h at 37 °C in 5% CO_2_ rich incubator, then 10 μM H_2_DCF-DA dye (100 µL) was added for an hour. The fluorescence intensity which correlates to ROS production was measured on a microplate reader at 485 and 535 nm excitation and emission, respectively. Data obtained were subjected to analysis using GraphPad Prism (version 8.2). 

*Statistical analysis*

Experiments were performed in triplicate and analyzed using a microplate reader (Varioskan Flash, Thermo Fisher Scientific, Vantaa, Finland). Results were presented as means ± standard deviation (SD). One-way ANOVA and Duncan multiplication range test was used to differentiate between means. Graphs and IC_50 _values were calculated using the GraphPad prism software (version 8.2). ^**^p < 0.01; ^***^p < 0.001. was considered significant.

## Results and Discussion

*Antimicrobial susceptibility test*

**Figure 1:**
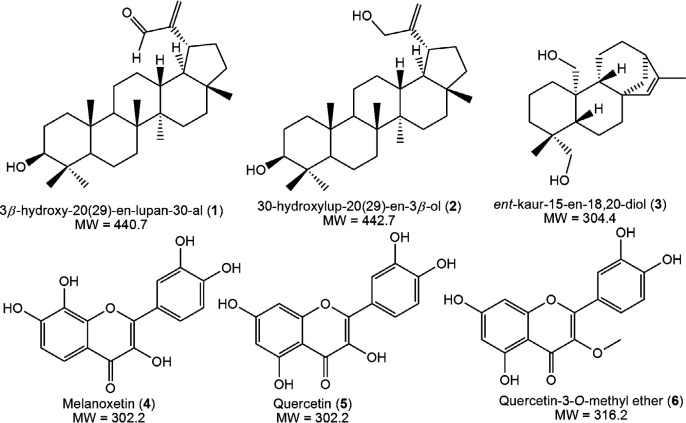
Chemical structures and molecular weight (MW) of compounds **1**-**6** isolated from *S. nigrescens* (adapted from Bodede *et al*., 2018 (11)).

The antimicrobial activity of compounds **1**-**6** (Figure 1) obtained from *S. nigrescens* tested over the concentration range 7.81 - 1000 µg/mL exhibited varying minimum inhibitory activity as shown in Table 1. Compounds showed MIC values ranging from 31.25 - 1000 µg/mL and for this study, MIC values that are not greater than 10-fold the antibiotic control were not considered significant. Compounds **5** and **6** showed good activity followed by compounds **4** and **3**. However, compound **3** had an insignificant MIC of 250 µg/mL against *E. coli*. Compounds **1** and **2** had MIC values ≥ 250 µg/mL across the three strains. 

Both lupane-type triterpenes (**1 **and **2**) had the same MIC values across the strains indicating that the difference in position 30 substitution (-CHO and -CH_2_OH) did not impact the antibacterial and antifungal activity of the compounds. Better activities were observed for the *ent*-kaurene (**3**) in *S. aureus* (MIC: 125 µg/mL) and *C. albicans* (MIC: 62.5 µg/mL). This may be due to the presence of a bicyclic system in the kaurenoid skeleton and lower molecular weight in comparison to triterpenes **1** and **2**. Earlier, it was reported that the flavonols **4**, **5,** and **6** had promising antibacterial and anti-quorum sensing activities which in this study was extended to the bacterial strain, *S. aureus* ATCC 25923, and the fungal strain, *C. albicans* ATCC 10231. Thus, the antimicrobial spectrum of **4**, **5,** and **6** may include antifungals in addition to their bacterial quorum sensing inhibition potential. 

Overall, all tested samples showed higher MIC against *E. coli* compared to *S. aureus* and *C. albicans*, which agrees with the notion that gram-negative bacteria like *E. coli* are more resistant than their positive counterparts.

*Cytotoxicity*

**Figure 2:**
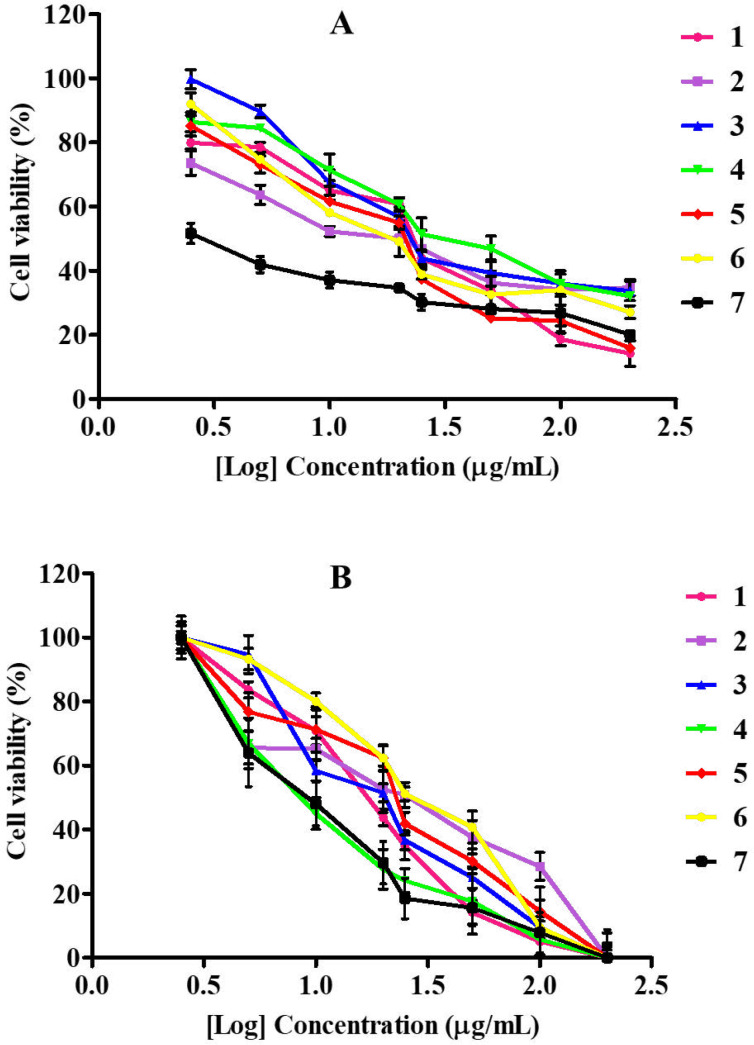
Cytotoxicity of *S. nigrescens* compounds on breast cancer cells (MDA-MB-231) (A) and RAW264.7 cells (B). Compound names: **1** - 3*β*-hydroxy-20(29)-en-lupan-30-al, **2** - 30-hydroxylup-20(29)-en-3*β*-ol, **3** - *ent*-kaur-15-en-18,20-diol, **4** - melanoxetin, **5** - quercetin, **6** - quercetin-3-*O*-methyl ether, **7** - doxorubicin. Data represent at least three experiments, each with n = 3 per group. The goodness of fit value R^2^ > 0.900

Cytotoxicity evaluated with the MTT assay on cancerous (MDA-MB-231) and non-cancerous (RAW264.7) cell lines revealed a dose-dependent effect when treated with pure compounds isolated from *S. nigrescens* with a concentration range of 200 - 2.5 µg/mL for 24 h. Tested concentrations showed no statistical differences between compounds treated cells and positive control, doxorubicin (Figure 2A). On the breast cancer cells, compounds **1**, **4** and **5** had IC_50_ values of 26.65, 22.17 and 20.61 µg/mL, respectively, while those of **2** (11.86 µg/mL), **3** (12.62 µg/mL) and **6** (14.03 µg/mL) were found to be comparable to the IC_50_ value of doxorubicin (9.35 µg/mL) (Table 2). For anticancer activity, IC_50_ values below 15 µg/mL are considered significant as they had 50% mortality of the cell population at a lower concentration (Table 2). An earlier cytotoxicity study on *A. mellifera* derived lupane-type triterpenes revealed that at least, presence of one hydroxyl group is required for expressing activity (18), which may be responsible for the observed activities of **1** and **2** in the present study. However, the *α*,*β*-unsaturated alcohol system on **2** had a better influence on cytotoxicity compared to *α*,*β*-unsaturated aldehyde system on **1**. The new *ent*-kaurene (**3**) also showed significant activity which may be due to the presence of two hydroxyl groups as observed for **2**. Sarwar *et al.* (2020) recently unveiled the molecular targets and mechanistic pathways of *ent*-kaurenes with significant anticancer potential (19). *Ent*-16β-17α-dihydroxykaurane (DHK), with a molecular weight of 306.4 had the most structural similarity to (**3**). Amongst the flavonols, quercetin-3-*O*-methyl ether (**6**) had the highest cytotoxic effect on MDA-MB-231 which is consistent with a recent study that confirmed its potency against the triple-negative breast cancer through extensive cytotoxicity, apoptosis and mechanistic studies (20). Although quercetin (**5**) was initially identified as a potential anticancer candidate (21, 22), further research brought to light its limitations (such as poor water solubility, bioavailability, easy oxidation, and toxicity to normal cells) (23) and its analogs and nano-hybrids subsequently developed, presented improved activities and few side effects (23, 24). It has been established that quercetin reaches the bloodstream in the form of different bio-transformed species including the methylated analogs (25). Thus, the better result of **6** compared to **5** makes the 3-*O* methyl derivative preferred over quercetin being the expected bioavailable species under *in-vivo* conditions. 

To evaluate the compounds’ selective cytotoxicity, the compounds were tested on RAW264.7, which are normal immune cells. All compounds affected cell viability in a dose-dependent manner (Figure 2B) with their corresponding IC_50_ values presented in Table 2. All compounds had low IC_50_ values, with **2**, **5** and **6**, having 22.81, 22.61, 28.73 µg/mL, respectively. The IC_50_ values of compounds **1**, **3** and **4** were 16.89, 18.50, 15.80 µg/mL, respectively, while the positive control was more toxic with an IC_50_ value of 10.61 µg/mL. Upon comparison of the compounds’ toxicity on RAW264.7 to the cancer cells, **1** and **4** were more toxic to the normal cells than the cancer cells. The triterpene **2** showed better selectivity (SI, 1.92) than the diterpene **3 **(SI, 1.46), whereas the well-known quercetin (**5**) had low selectivity with SI (1.09) comparable to the control, doxorubicin (1.13). These findings suggest that the *α*,*β*-unsaturated alcohol moiety of **2** is preferable to *α*,*β*-unsaturated carbonyl moiety of **1** for selective cytotoxicity. The positions of hydroxy substitutions on the flavonol skeleton probably play a significant role in the selectivity observed across **4**, **5** and **6**. Compound **6**, the 3-*O* methyl ether derivative of quercetin was most selective to the carcinoma cells with the highest selectivity index of 2.04. The toxicity of bioactive compounds to non-cancerous cells remains a great concern in cancer chemotherapy. More work is still required on the modification of plant-derived therapeutic agents for better cytotoxic selectivity.

*Intracellular ROS production in RAW264.7*

**Figure 3:**
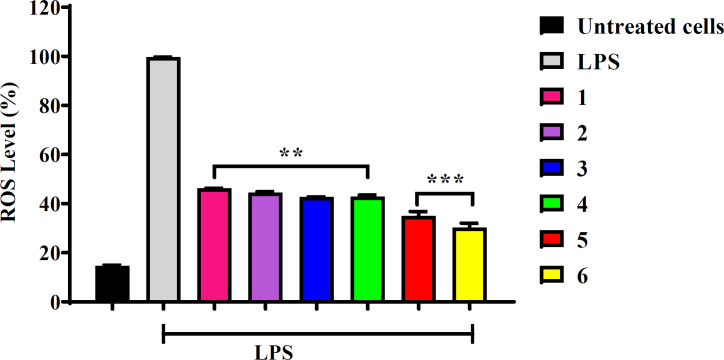
Effects of *S. nigrescens* compounds **1**-**6** on LPS-induced ROS production by RAW264.7 cells measured by a micro plate reader (Thermo Scientific Varioskan Flash). Cells were pre-treated with compounds (10 µg/mL) for 20 h and stimulated with LPS (10 µg/mL) for 4 h. Experiments were conducted in triplicate and data is expressed as the mean ± SD with *p*-values calculated against control ^**^p < 0.01; ^***^p < 0.001

Inflammation is a physiological response stimulated by the organism as a way of overcoming pathogenic events employed as a defense mechanism. However, an unregulated inflammatory response can lead to several diseases, including cancer. ROS-inducing approaches rely on the fact that increasing the ROS level over the cytotoxic threshold can result in cellular impairment, damaging DNA, RNA, proteins and lipids in normal cells, even though macrophage cells cannot phagocytize cancerous cells, but release a reasonable amount of pro-inflammatory cytokines that quench cancer cells yet are cytoprotective to normal cells (6). In this regard, it was considered that the compounds tested would be able to reduce the production of LPS-induced ROS in normal macrophages. Determination of ROS production in RAW264.7 cells was conducted using the non-toxic IC_50_ value of 10 µg/mL for all compounds (Table 2). Cells were pre-treated with compounds for 20 h and LPS was added in all treated cells except the untreated control cells. Thereafter H_2_DCFDA was introduced to the cells. As observed in Figure 3, all tested compounds significantly decreased the ROS production compared to the LPS-treated cells. Due to high metabolic activity, cancer cells produce high levels of ROS to evade immune anticancer response. However, the continuous elevation of ROS from cancer cells infiltrates immune cells by inducing oxidative stress. Therefore, the basis of conducting this investigation on normal (Raw264.7) cells was to assess the protective power of the compounds against ROS toxicity, which leads to oxidative stress and cells death. However, the possible mechanism of ROS reduction effect in cells treated with compounds might be associated with the direct free radical scavenging activity or indirect protection from oxidative stress.

Flavonoids and terpenoids are known for their antioxidant and anti-inflammatory properties (26-29). Quercetin and related flavonols have shown potentials as anti-inflammatory agents. Kim *et al.* (2004) reported that quercetin down-regulated the release of LPS-induced pro-inflammatory mediators including IL-1, IL-6, TNF-α in RAW 264.7 cells (30). Recently, it was found that quercetin significantly reduced ROS intracellularly, thereby protecting L02 cells from D-galactosamine (D-GaLN)-induced damage (31). The present study demonstrated that quercetin **(5) **and its 3-*O*-methyl ether **(6)** significantly reduced ROS production to levels below 50% (Figure 3), which agrees with previous findings. However, contrasting reports exist where quercetin and similar flavonols upregulated the LPS-induced release of proinflammatory mediator IL-6 and IL-8 as opposed to other flavonoid groups like flavones, isoflavones, flavanes and chalcones which reduced the same interleukins (IL-6 and IL-8) (32). Melanoxetin **(4)** and the terpenoids** (1–3)** tested in this study exhibited approximately 40% reduction of ROS. Pharmacological studies of the phenolic compounds in *Acacia confusa* revealed that **4** is a strong inhibitor of LPS-induced ROS and RNS with IC_50_ values of 12.5 μM and 6.9 μM, respectively (33, 34).

Mediation of oxidative stress by regulating pro-inflammatory cytokines initiates the lipid peroxidation process and may lead to the damage of bacterial/cell membrane, thereby proposing a possible mechanism of antimicrobial and anticancer activity. To support this view, studies conducted on the antimicrobial mechanisms of action of flavonoids proposed mechanisms such as inhibition of nucleic acid synthesis, interruption of the cytoplasmic membrane functions and disruption of energy metabolism (35, 36). However, the mechanisms are not limited to flavonoids but include terpenoids (37). Resveratrol and quercetin reduced the nitric oxide (NO) production in *Salmonella typhimurium* infected human myeloid leukemia cell line (U937 cells), as a result, cell viability and proliferation in infected cells were inhibited. Moreover, the compounds showed protective effects of the host cells from the toxic effects of bacterial infection and decreased programmed cell death (38). Furthermore, quercetin and quercetin-3-*O*-rhamnoglucoside exerted their antimicrobial activity by reducing the bilayer thickness of microorganisms (39). It is also likely that the *in vitro* microenvironment of quercetin and its derivatives (as seen in the present study), creates a redox condition that permits a protective ROS inhibition or pro-oxidant antimicrobial function. Apigenin-8-C-glucoside was shown to exhibit antimicrobial activity against *S. aureus* and its mechanism was suggested as reducing the hydrophobicity of cell surface and membrane permeability at an MIC = 126 µg/mL (40).

## Conclusion

Six *S. nigrescens* compounds exhibited strong biological activities when evaluated for their antimicrobial, cytotoxicity, and reactive oxygen species inhibitory capacity. Generally, compounds **5 **and **6** showed strong antimicrobial activity and good activity in reducing cancerous viable cells. All compounds suppressed the LPS-induced ROS levels. Our findings further justify the ethnomedicinal use of *S. nigrescens* whose phytoconstituents possess remarkable therapeutic potentials. The antimicrobial activity of the compounds could be extrapolated to the plant’s potential for treating wounds and skin diseases. The low cytotoxic selectivity of the compounds suggests that care must be taken in the therapeutic usage of single-agent plant-derived metabolites. Prospective studies should be done to understand the mechanism of action especially investigating the regulation of mitogen-activated protein (MAP) kinases and nuclear factor (NF)-κB cascades and proinflammatory mediators including TNF-α, interleukins (IL), and MIP-2 secretion by compounds tested in this study.

## Author’s contributions

Conceptualisation: Olusola Bodede 

Investigation and data curation: Garland K. More and Olusola Bodede

Project administration and supervision: Gerhard Prinsloo

Writing, review, editing and approval of final manuscript: All authors

## Conflicts of interest

The authors declare no conflict of interest.
